# Effect of flexing deformations on functional properties of laminated silica aerogel fibrous matting composites for footwear applications

**DOI:** 10.1186/2046-7648-4-S1-A69

**Published:** 2015-09-14

**Authors:** Polona Kraner Zrim, Igor B Mekjavic, Tatjana Rijavec

**Affiliations:** 1 Faculty of Natural Sciences and Engineering, Department of textiles, University of Ljubljana, 1000 Ljubljana, Slovenia; 2 Institut Jožef Stefan, Department of Automation, Bicyberneticts and Robotics, Ljubljana, Slovenia

## Introduction

Silica-aerogel composite, a silica-aerogel reinforced with fibrous matting, are being considered as an isolative material for footwear. The suitability for such applications has been investigated in the study presented in this article. Experiments were conducted on laminated silica aerogel composite (LAC). Lamination of silica aerogel composite with a solid membrane is needed to prevent spreading of crushed silica aerogel dust into surrounding. LAC was subjected to cyclic of flexing to simulate mechanical stress that occurs during normal usage condition in footwear applications. The study highlights the impact of the irreversible crushed structure of silica aerogel on the permeability and thermal resistance, tensile properties and the quality of lamination.

## Methods

*Cyclic flexing *was performed on a Bennewart machine, originally used for testing shoe sole materials. It was performed in accordance with ISO 17707:2005 (E) standard that illustrates test methods for personal protective equipment and footwear. Thermal resistance was measured on a single sample heat flow meter apparatus with symmetrical configuration in accordance to standard EN 12667:2002. *Water vapor permeability *was determined according to EN 13515:2001, which is used for footwear uppers and lining materials and is appropriate for thicker materials. *Tensile strength*, of materials was measured on a dynamometer Instron 5567 (Instron, GB) according to standard ISO 13934-1. *Delamination test*, was performed on a dynamometer Instron 5567 (Instron, GB) in accordance with standard EN 15619:2008.

## Results

See Tables 1 and 2.

**Table 1:** 

	Thermal resistance [m²K.W^-1^]	Water vapour permeability [mg. cm^-2^.h^-1^]	Tensile strength [MPa]		Mean force at delamination [N]
			**Longitudinal**	**Transverse**	

LAC	0.1861 (0.010)	1.3 (0.07)	3.40 (0.18)	2.40 (0.11)	7.3 (0.7)

LAC after flexing	0.1838 (0.007)	1.3 (0.06)	2.84 (0.05)	1.86 (0.1)	6.0 (0.8)

**Table 2:** 

	Thermal resistance [m²K.W^-1^]
Thinsulate	0.0661 (0.001)

Polyurethane foam laminate	0.0822 (0.002)

## Discussion

It was found that flexing caused crushing of the silica aerogel and loosening of fibrous structure of the composite but did not damage the membrane on the laminated composite. No statistically significant differences of thermal resistance and water vapor permeability could be measured between unflexed and flexed samples. The delamination test showed that a 17.8 % lower mean force was needed to delaminate flexed samples. A decrease of tensile strength of 16.4 % was measured in the longitudinal direction and of 21.9 % in the transverse direction of flexed samples.

## Conclusion

Flexing had no effect on the thermal resistance and water vapor permeability of the LAC. It increased the tendency of laminated silica aerogel composite to delaminate and deteriorated its tensile properties but in a range where that had no effect on the end use. Compared to LAC the thermal resistance (at a thicknesses that are comparable to that of LAC) of Thinsulate is 63 % lower and that of laminates with polyurethane foams is 54 % lower. The study confirmed that the newly developed laminate has potential applications in apparel and footwear for extreme temperature environments.

